# Changes in secondhand smoke exposure levels and risk of type 2 diabetes in middle age: the Korean Genome and Epidemiology Study (KoGES)

**DOI:** 10.1136/bmjdrc-2019-000859

**Published:** 2019-12-08

**Authors:** Jooeun Jeon, Keum Ji Jung, Heejin Kimm, Sun Ha Jee

**Affiliations:** 1 Department of Public Health, Graduate School, Yonsei University, Seoul, South Korea; 2 Department of Epidemiology and Health Promotion, Institute for Health Promotion, Yonsei University Graduate School of Public Health, Seoul, South Korea

**Keywords:** smoking, adult diabetes, longitudinal study, epidemiology

## Abstract

**Objectives:**

Secondhand smoke (SHS) was known as one of the risk factors for type 2 diabetes. So far, some studies revealed the association of SHS exposure and type 2 diabetes, however, no studies to show the relationship of cumulative SHS exposure with type 2 diabetes exist. Therefore, the objectives of this study were to identify subgroups of participants who share similar trajectories in SHS exposure levels in middle age by using latent class growth modeling, and determine the independent association of these SHS exposure level trajectories with risk of incident type 2 diabetes.

**Methods:**

In Korean Genome and Epidemiology Study (2001–2014), 2079 participants aged 40 years and above who received biennially health check-up to follow-up and with available information of SHS exposure were selected. Four distinct trajectory groups (low-stable, moderate to low, moderate, and high to low) were identified for SHS exposure levels using trajectory modeling methods. Multivariable Cox proportional hazards model was used to examine the association of trajectories with risk of type 2 diabetes.

**Results:**

During 24 083.3 person-years of follow-up (mean follow-up duration, 11.6 years), 200 incident cases of type 2 diabetes and 640 incident cases of impaired fasting glucose (IFG) were identified. In multivariable Cox model, ‘High to low’ trajectory was significantly associated with risk of type 2 diabetes (OR 1.9; 95% CI 1.3 to 2.8) compared with ‘Low-stable’. For IFG, all trajectories had significantly 30%–30% higher risk of type 2 diabetes compared with the ‘Low-stable’ trajectory.

**Conclusions:**

Changes in SHS exposure levels have been shown to associate with subsequent type 2 diabetes risk. Reversing high exposure level of SHS in middle-aged adulthood may still lead to worse progressions of type 2 diabetes than remaining stable exposure level.

**Significance of this study**

**What is already known about this subject?**

- Secondhand smoke (SHS) is known as one of the risk factors of type 2 diabetes as well as smoking.
- There were already some meta-analyses assessing the effect of SHS on type 2 diabetes risks, and dose–response relationship.

**What are the new findings?**

- Distinct SHS trajectories over time were identified under life course approach.
- Changes in SHS exposure levels over time may contribute to development of type 2 diabetes.
- Even reversing the high level of SHS in middle-aged adulthood can still worsen the progress of type 2 diabetes due to cumulative effects.

**How might these results change the focus of research or clinical practice?**

- Our study showing the association between cumulative SHS effect and worsening of glucose metabolic structure prompts the need for more reliable and cost-effective methods for interventions to prevent secondhand smoking.
- Our study can highlight the importance of SHS exposure prevention across life span, and prognostic assessments and targeted strategies for high-risk individuals based on the trajectory modeling.

## Introduction

Diabetes is the major enemy threating global health.^1 2^ Also, the global economic burden of diabetes is increasing continuously.^3^ Recently, the number of people with pre-diabetes is increasing sharply in the USA.^4^ In the aftermath of the ageing population in Korea, also, the prevalence of type 2 diabetes among adults 30 years or older is 14.4% and approximately 3 out of 10 adults above 65 years old have diabetes in Korea in 2016.^5^

Until now, some researchers are interested in the issue, secondhand smoke (SHS) exposure and chronic diseases. Also, some epidemiological studies have raised the relevance between SHS exposure and type 2 diabetes,^6–13^ and have revealed the existence of an association as well as the dose–response relationship.^14^ However, previous studies were conducted with data based on elderly population,^6^ and studied with single variable about whether exposure of SHS or not.^8 15 16^ Only two studies with variable for levels of SHS exist, however, these studies have limitations to generalization because of studying on child and women and without cumulative risk.^9 10^

Recently, the life course approach concept is raised up in public health, and most studies on this approach use trajectory modeling to identify cumulative or changeable risk patterns.^17^ This approach can bring new insight up that risk factors can be mobile and there are differences in chronic disease-onset points dependent on each individual health status.^18^ Until now, a dose–response relationship between SHS exposure and type 2 diabetes was found in previous studies,^14^ however, the harmful effects of SHS exposure in real-world by life course approach were not found. Therefore, the objectives of the present study were to apply trajectory models to identify distinct trajectories of SHS exposure in Korean adults and to examine the relationship between SHS exposure trajectories and type 2 diabetes incidence.

## Methods

### ​Study population

**Figure 1:**
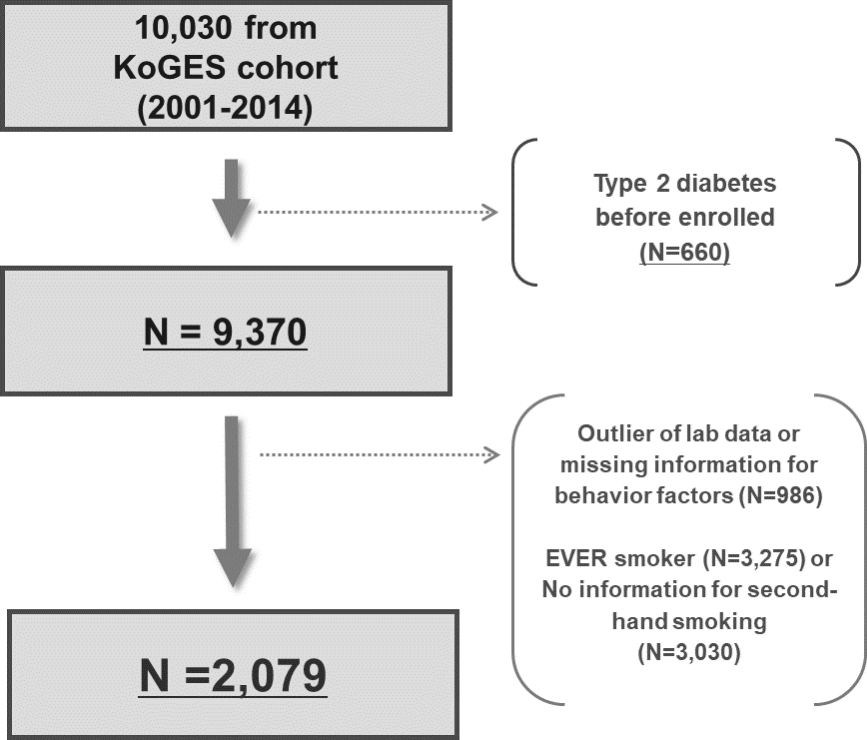
Flowchart for selection of the study population. KoGES, Korean Genome and Epidemiology Study.

Base data were obtained from KoGES_Ansan and Ansung study, which is based on the Korean Genome and Epidemiology Study (KoGES), a seven-wave longitudinal data set that included 10 030 Korean adults aged 40 years or more based on baseline (2001–2002), and received biannually health check-up to follow-up since baseline recruitment in baseline (2001–2002) up to the sixth follow-up. Out of 10 030 baseline participants, 660 individuals with diagnosis of type 2 diabetes, 3275 individuals who were ever smoker, 3030 individuals who had no information for SHS, and 986 individuals with missing information or outlier of covariates were excluded. Therefore, a total of 2079 participants (281 men and 1798 women) were included in the final sample (figure 1).

### ​SHS exposure levels

Participants of KoGES underwent biannually health check-up with a standard questionnaire by trained research staff for this cohort under the manual from baseline (2001–2002) to endline (wave 6; 2013–2014). A standard questionnaire of KoGES includes whether exposure of SHS or not as well as detail questions for SHS: frequency of SHS (0: none, 1: <3 days per/week, 2: ≥3 days/week, 3: every day) and SHS time (min/day). Measurements of SHS time (min/day) from each wave were used to apply to latent class growth modeling (LCGM) in this study.

### ​Ascertainment of diabetes and follow-up

Type 2 diabetes was defined based on a self-reported current treatment with antidiabetes medication and/or as fasting glucose ≥126 mg/dL and/or glycated hemoglobin (HbA1c) ≥6.5% by KoGES (Ansung-Ansan) cohort’s questionnaire. For more detailed information on KoGES (Ansung-Ansan) cohort’s questionnaire, we could confirm through the previous cohort profile publication for this cohort.^19^ Follow-ups for type 2 diabetes cases were conducted from baseline (2001–2002) to onset of type 2 diabetes. Follow-ups for all participants without the onset of type 2 diabetes were conducted from baseline (2001–2002) to 31 December 2014.

### ​Measures of covariates

Height and weight were obtained by trained research staff in the KoGES (Ansung-Ansan) cohort study. Body mass index (BMI) using height and weight is the most typical method to estimate fat. It is measured as weight (in kilograms) divided by square of height (in meters). BMI was calculated from these measured values (height and weight at baseline) by this cohort follow-up procedure according to the accepted formula.

The following self-reported variables were used as categorical variables: family history of type 2 diabetes (yes, no), alcohol drinking status (non-drinker, ex-drinker, and current drinker), regular exercise (regular exercise, and no exercise) and educational levels (below middle school, high school, some college, and above graduate school), and household income levels (<1 million won, 1 to <2 million won, 2 to <4 million won, and >4 million won). Physical and laboratory examinations were conducted to collect clinical variables. Age, sex, family history of type 2 diabetes, alcohol drinking status, regular exercise, BMI, systolic blood pressure (SBP), total cholesterol, educational levels, and household income at baseline were incorporated as covariates in the present study.

### ​Statistical analysis

#### ​Latent SHS exposure trajectory identification

Heterogeneity in the longitudinal development was investigated using LCGM to identify distinct subgroup of participants’ SHS exposure levels over follow-up time.^20^ Each individual who has included subgroups shares similar underlying SHS exposure trajectories during the follow-up period.^21^ The longitudinal nature of data was modeled by having the parameter depending on time. Time-stable covariates (risk factors) were incorporated into the model by assuming that they could influence the probability of belonging to a particular group.^20^ The distinct groups of SHS exposure were formed using Bayesian information criteria (BIC) to determine the best number of distinct groups (BIC=log (likelihood)−0.5*log (sample size)*−number of parameters). Models were estimated with three to seven trajectories by assuming linear, quadratic, and cubic patterns of change in SHS exposure levels over time using SAS PROC TRAJ package (SAS Institute, Cary, NC, USA). The best model was automatically selected considering class membership posterior probabilities (the highest) with the lowest absolute BIC value. To ensure that all obtained classes were of clinically statistically meaningful size, the condition that each class should include at least 5% of participants and the mean posterior probability of each class should be higher than 75% was imposed.^22^

Finally, four distinct trajectory groups of SHS exposure were identified. They were labeled according to their specific patterns (‘Low-stable’, ‘Moderate to low’, ‘Moderate’, ‘High to low’). After obtaining trajectories, within each identified SHS exposure group, trajectories of change in other related risk factors during the follow-up were developed and independent effects of SHS exposure trajectories on risk of type 2 diabetes by person-years per 100 000 were assessed.

#### ​Inverse probability of censorship weighting

To correct for bias from loss to follow-up, an inverse probability of censorship weighting (IPCW) approach was used in this present study. At the first stage, we set newly an indicator variable for loss to follow-up among all participants in the final sample (n=2079) in order to identify those with data at baseline who did not contribute to the final follow-up visit. And then a multivariable logistic regression model was formed with a new indicator variable as the dependent variable (1=remained in this study; 0=lost to follow-up). Age, sex, alcohol drinking status, physical exercise, BMI, SBP, total cholesterol level, educational levels, and household income were used as independent variables in this model. The predicted probabilities (p) from the logistic model involved means the probability remaining in the study, and inverse probability of censorship weights were calculated as 1/p.^18 23^

#### ​Association of SHS exposure trajectory groups with type 2 diabetes incidence

Subsequently, the multivariable Cox proportional hazards model with weight was fitted after adjusting for covariates at baseline to quantify the association between SHS exposure trajectories and type 2 diabetes risk. We additionally analyzed to estimate the HR risk of type 2 diabetes according to trajectories of SHS exposure among women to confirm gender difference. We also estimated the interaction effect for type 2 diabetes risk between SHS trajectories and gender by using the multivariate Cox proportional hazards model added type 3 option with Likelihood Ratio (LR) command, which can test based on a likelihood ratio test to calculate p values for interaction.

All analyses were performed using SAS statistical software V.9.4 (SAS Institute). All other statistical tests were two sided and statistical significance was determined as p<0.05.

## Results

**Figure 2:**
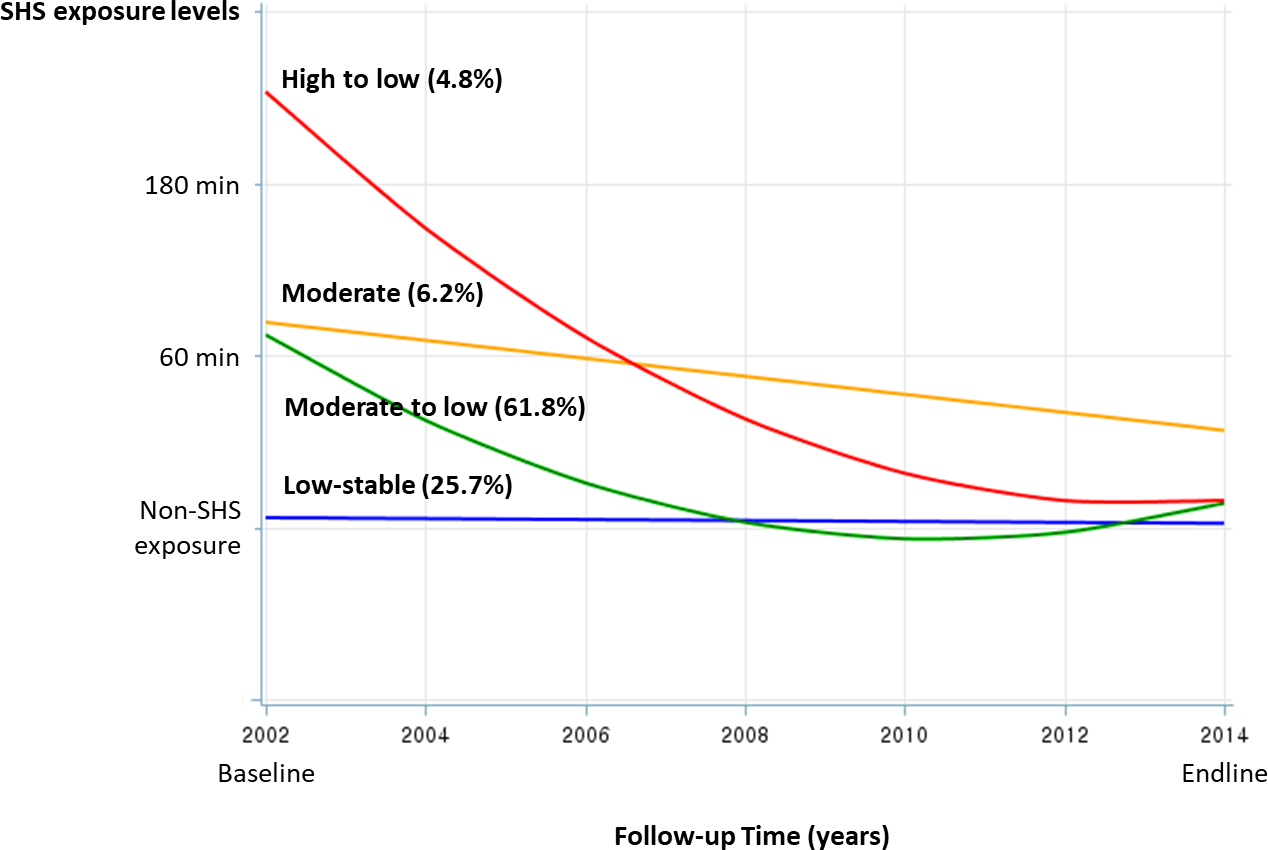
Changes in secondhand smoking exposure levels during follow-up period by trajectory groups. SHS, secondhand smoke.

From trajectory modeling, we identified four SHS exposure trajectories, which were significantly unique from the others at the p<0.0001 level. BIC scores for the number of groups were −9972.54 in the best trajectory model. The percentage of participants of each of the four trajectories ranged from 4.8% to 49.0% (figure 2). In this result from trajectory modeling, we found that ‘High to low’ trajectory was shown rapidly decreasing pattern of SHS exposure level though the highest exposure level at baseline.

Table 1 shows the mean of exposure level at each time to follow-up points according to four SHS exposure trajectories. ‘Low-stable’ trajectory steadily stayed around 20–30 min/day exposure level of SHS during the follow-up period. On the other hand, ‘High to low’ trajectory was shown the highest level of SHS exposure as up to about 300 min/day at baseline. However, at the endline (finished point of follow-up), it showed similar exposure levels per day to ‘Low-stable’ trajectory.

**Table 1:** Mean of exposure time at each study examination for secondhand smoking exposure trajectories, n=2079

n	Low-stable	Moderate to low	Moderate	High to low	P value*
	534	1285	128	132	
	Mean±SD				
Exposure levels (min/day)					
0 year (baseline)	29.8±27.6	65.79±126.2	77.3±97.4	295.2±277.8	**<0.0001**
2 years	21.9±21.4	45.8±83.7	62.8±76.4	227.4±244.3	**<0.0001**
4 years	30.1±39.8	21.5±26.1	36.9±55.7	78.5±125.0	**<0.0001**
6 years	35.1±47.2	17.8±27.0	32.4±66.0	60.7±98.0	**0.0002**
8 years	15.8±18.0	19.3±26.2	37.5±57.9	56.0±80.9	0.1362
10 years	22.5±23.6	12.3±13.4	35.6±52.8	45.8±51.0	**0.0254**
12 years (endline)	26.2±22.1	28.9±44.4	38.1±52.4	33.1±55.9	0.5368

*The bold values are statistically significant.

SHS, secondhand smoke.

A total of 2079 participants as a final sample contributed 24 083.3 person-years of follow-up time (mean=11.6, SD=2.3), and a mean age of years at baseline was 50.5. Out of them, women had a higher proportion (86.5%) than men (13.5%). Also, except ‘Low-stable’ trajectory, almost women were included in the other three trajectories. There were no differences across four SHS trajectories for BMI, fasting glucose, SBP, and total cholesterol level at baseline (table 2). However, at the finished line to follow-up, there were differences across four SHS trajectories for SBP and total cholesterol levels. For behavior factors (alcohol drinking status and regular exercise) or socioeconomic status (educational levels and household income), there was a difference. Especially, the ‘High to low’ trajectory was shown a higher proportion of participants who had low educational levels or low household income (table 2).

**Table 2:** Participant’s general characteristics at baseline in Korea (KoGES), n=2079

n	Low-stable	Moderate to low	Moderate	High to low	P value
	534	1285	128	132	
	**Mean±SD**				
Age (years)					
At baseline	49.03±7.64	51.40±8.80	49.68±8.15	49.49±7.74	**<0.0001**
BMI (kg/m^2^)					
At baseline	24.79±2.88	24.84±3.22	25.46±3.44	24.96±3.87	0.3322
At endline*	24.53±2.90	24.67±3.23	25.34±3.62	25.17±4.12	0.0641
Fasting glucose (mg/dL)					
At baseline	89.23±10.85	87.80±10.87	87.71±10.67	87.70±3.87	0.0505
At endline	94.86±15.73	94.24±16.60	97.41±21.67	87.60±8.76	0.1468
Systolic BP (mm Hg)					
At baseline	117.98±18.28	119.75±20.12	121.94±21.40	120.65±19.32	0.2002
At endline	118.44±15.96	120.94±18.34	125.12±17.29	124.07±20.40	**0.0013**
Total cholesterol (mg/dL)					
At baseline	197.14±34.58	197.73±36.43	191.19±31.53	194.65±34.25	0.1631
At endline	185.83±33.48	190.70±34.58	184.08±29.41	182.59±35.92	**0.0196**
** **	**%**				
Sex, women	53.93	97.43	98.44	100.00	**<0.0001**
Current drinker	49.55	31.22	26.87	28.68	**<0.0001**
Regular exercise	37.48	29.01	27.61	25.74	**0.0002**
Educational levels					
Up to middle school	23.60	43.19	41.41	43.94	**<0.0001**
High school	24.72	23.74	32.81	28.03	**<0.0001**
Some college	35.77	27.78	27.78	23.48	**<0.0001**
At least graduate school	15.92	5.29	5.29	4.55	**<0.0001**
Household income, 10 000 won					
<100	23.60	37.82	47.66	48.48	**<0.0001**
100–199	28.65	30.51	28.13	35.76	**<0.0001**
200–399	36.52	25.91	17.97	20.45	**<0.0001**
400+	11.24	5.76	6.25	5.30	**<0.0001**

The bold values are statistically significant.

*The number of sample was 1318 after excluding participants who had no body mass index value at the endline.

BMI, body mass index; BP, blood pressure; KoGES, Korean Genome and Epidemiology Study; SD, standard deviation; SHS, secondhand smoke.

Results for the risk of diabetes HR for each SHS exposure level trajectory with ‘Low-stable’ trajectory as a reference are shown in table 3. During 24 083.3 person-years of follow-up (mean follow-up, 11.6 years), 200 incident cases of type 2 diabetes and 640 incident cases of impaired fasting glucose were documented. Age-standardized incidence rates of type 2 diabetes were increased with SHS exposure levels: 904.9 in the ‘Low-stable’ trajectory, 700.8 in the ‘Moderate to low’ trajectory, 880.5 in the ‘Moderate’ trajectory, and 1728.5 in the ‘High to low’ trajectory. Also, for impaired fasting glucose, age-standardized incidence rates were increased with SHS exposure levels: 2922.9 in the ‘Low-stable’ trajectory, 2684.1 in the ‘Moderate to low’ trajectory, 4402.2 in the ‘Moderate’ trajectory, and 2938.6 in the ‘High to low’ trajectory.

**Table 3:** RRs (95% CI) for risk of type 2 diabetes according to trajectories of secondhand smoking exposure levels, n=2079

	T2DM incidence, n	Person-years, follow-up	Age-adjusted rate	Unadjusted model	Multivariable model*	Fully adjusted model†
				RR (95% CI)	RR (95% CI)	RR (95% CI)
Trajectories of secondhand smoking exposure						
Low-stable	51	6155.20	904.92	1.00	1.00	1.00
Moderate to low	113	14 993.13	700.81	0.94 (0.74 to 1.20)	0.74 (0.56 to 0.98)	0.99 (0.71 to 1.37)
Moderate	12	1479.73	880.46	1.02 (0.66 to 1.57)	0.80 (0.50 to 1.27)	0.65 (0.37 to 1.15)
High to low	24	1455.20	1728.51	**2.01 (1.42 to 2.83**)	**1.91 (1.31 to 2.78**)	**1.92 (1.22 to 3.04**)
P for trend				**0.0009**	**0.0016**	**0.0453**

The bold values are statistically significant.

*Model adjusted for age, sex and family history of type 2 diabetes, alcohol drinking status, physical exercise, and body mass index, systolic blood pressure, total cholesterol level, educational levels, and household income at baseline.

†Model adjusted for multivariable model+body mass index, systolic blood pressure, and total cholesterol level at endline.

RR, risk ratio; T2DM, type 2 diabetes mellitus.

In the unadjusted model, the ‘High to low’ trajectory had a significantly higher risk of type 2 diabetes compared with the ‘Low-stable’ trajectory (HR=2.0, 95% CI 1.4 to 2.8). In multivariate Cox proportional hazards model after adjusting for age, sex and family history of type 2 diabetes, BMI, behaviors, biological indicators and socioeconomic status (alcohol drinking status, physical exercise, SBP, total cholesterol level, educational levels, and household income), the ‘High to low’ trajectory had significantly higher risk of type 2 diabetes (HR=1.9, 95% CI 1.3 to 2.8). After additionally further adjusting for BMI, SBP, and total cholesterol level et endline, ‘High to low’ trajectory had a significantly higher risk type 2 diabetes compared with the ‘Low-stable’ trajectory (HR=1.9, 95% CI 1.2 to 3.0). Meanwhile, for impaired fasting glucose (table 4), all trajectories had significantly higher risk compared with the ‘Low-stable’ trajectory after adjusting for covariates (HR=1.3, 95% CI 1.1 to 1.5 for moderate to low; HR=1.3, 95% CI 1.0 to 1.7 for moderate; HR=1.4, 95% CI 1.0 to 1.9 for high to low, respectively). There was no interaction between secondhand smoking and age, or sex (p=0.1810 for age, p=0.5646 for sex).

**Table 4:** RRs (95% CI) for risk of impaired fasting glucose according to trajectories of secondhand smoking exposure levels, n=2079

	T2DM incidence, n	Person-years, follow-up	Age-adjusted rate	Unadjusted model	Multivariable model*	Fully adjusted model†
				RR (95% CI)	RR (95% CI)	RR (95% CI)
Trajectories of secondhand smoking exposure						
Low-stable	166	5710.33	2922.91	1.00	1.00	1.00
Moderate to low	379	13 734.32	2684.10	0.98 (0.86 to 1.12)	**1.23 (1.04 to 1.44**)	**1.25 (1.05 to 1.49**)
Moderate	55	1262.62	4402.21	**1.55 (1.25 to 1.91**)	**1.89 (1.50 to 2.40**)	**1.31 (1.02 to 1.69**)
High to low	40	1402.04	2938.58	0.97 (0.76 to 1.24)	**1.33 (1.02 to 1.73**)	**1.39 (1.04 to 1.85**)
P for trend				**0.1590**	**0.0002**	**0.0147**

The bold values are statistically significant.

*Model adjusted for age, sex and family history of type 2 diabetes, alcohol drinking status, physical exercise, and body mass index, systolic blood pressure, total cholesterol level, educational levels, and household income at baseline.

†Model adjusted for multivariable model+body mass index, systolic blood pressure, and total cholesterol level at endline.

RR, risk ratio; T2DM, type 2 diabetes mellitus.

Sensitivity analyses were conducted to compare findings in women by rerunning all models. Results are shown in online supplementary tables 1 and 2. These results addressed similar aspects in women only for the risk of type 2 diabetes or impaired fasting glucose in all subjects. In the fully adjusted model, ‘High to low’ trajectory had a significantly higher risk compared with the ‘Low-stable’ trajectory (HR=1.8, 95% CI 1.1 to 2.8 for type 2 diabetes; HR=1.5, 95% CI 1.1 to 2.1 for impaired fasting glucose).

10.1136/bmjdrc-2019-000859.supp1 Supplementary data

## Discussion

The current study paid particular focusing on the application of trajectory modeling based on a life course approach for longitudinal data. In our population-based cohort study, middle-aged people were followed up biennially, and four different patterns of SHS exposure level trajectories for over 14 years were found by using trajectory modeling. The trajectory that developed the highest risk of type 2 diabetes had a rapidly decreasing trend for SHS exposure level over time and similar SHS exposure level at the finished point of follow-up compared within the reference trajectory that had stable on the lowest range during the follow-up time. This finding is meaningful as an aspect that even though resolving the external exposure of SHS in middle adult, elevated SHS exposure level up to above 180 min/day in early adulthood may alter glucose metabolic structure in a way that is not reversible. Also, despite the highest level of SHS exposure at the finished point of follow-up, the trajectory that showed slightly decreasing (‘Moderate’ trajectory) trend during the follow-up period from 60 to 70 min/day at baseline had no relationship with type 2 diabetes risk.

So far, many epidemiologists already have studied the issue of the association between smoking and type 2 diabetes.^12 24^ Also, the harmful effects of SHS exposure have been noted since 1928.^25^ However, when the Surgeon General Report was published in 1964, we did not know enough about the harmfulness of SHS exposure. And then some researchers have suggested the hazard of SHS intermittently through related previous studies.^26^

Indeed, there was a try for examining the association of SHS exposure and risk of type 2 diabetes among Koreans in a previous cohort study, which showed that participants exposed to SHS had a significantly increased risk of 1.41-fold for type 2 diabetes after adjusting for covariates.^7^ As regards the highest level of SHS exposure had the highest risk of type 2 diabetes, our findings are consistent with results from previous studies showing the dose–response relationship. Eze and his colleagues found the strong dose–response relationship, that subjects exposed to SHS as up to 3 hours/day in the home had a significant risk of 2.6-fold for type 2 diabetes; however, the previous result has the limitation of exposure site.^10^ Also, for impaired fasting glucose, all exposure trajectories had a 30%–40% higher risk of type 2 diabetes compared with a trajectory with the lowest level of SHS exposure. That finding supports the theory in previous studies that if we once had even little exposure level to SHS, we may affect from hazardous matters from SHS and defect our glucose metabolic structure.^7 12 14^

The biological effect of SHS exposure on fasting glucose levels could be extrapolated from the effects of typical smoking. Nicotine stimulates pharmacologically the sympathetic nervous system in active smokers and insulin-antagonizing substances such as cortisol, catecholamine, and growth hormone.^27^ Also, It may influence the development of diabetes as side effects of nicotine have been reported, including the inhibition of gastric mortality and its influence on the differentiated emptying of solids and liquids, faster glucose absorption, and increased erythrocyte permeability to glucose.^28–30^^31^ In addition, a recent study found that smoking by using electronic cigarettes can likely increase the type 2 diabetes risk in a general population.^32^

Meanwhile, SHS that contains more than 7000 chemicals has been known as a risk factor for diabetes from long time ago. According to the 1964 Surgeon General’s Report, 2.5 million adults who were non-smokers died because of their breathing SHS. Especially, SHS as forced smoking can harm children and adolescents in their home, school, and public places. In this present study, we revealed that even though resolving the external exposure of SHS in middle adults, if we once had exposure to SHS as about 180 min/day, damaged glucose metabolic structure from SHS is not reversible. Thus, we can suggest the most important point through our study is the segregation from smokers, and the only way to fully protect non-smokers or young people who do not smoke is to eliminate smoking in all homes, worksites, schools and public places.

This finding also further highlights that type 2 diabetes is a heterogeneous disease with different pathophysiological pathways unknown related to smoking. In our results of this study, the SHS trajectory (‘High to low’ trajectory), which showed a higher risk of type 2 diabetes than other trajectory and sharply decreasing level of SHS exposure, had patterns of fasting glucose level similar to other trajectory during follow-up period (online supplementary figure 1). However, we found the differences in BMI or SBP at endline across trajectories. As regards that, the trajectory, which had about twofold risk of type 2 diabetes, had a steadily stable trend of BMI value as the level of obesity over follow-up time (online supplementary figure 2). In general, most middle-aged people can obtain chronic disease such as type 2 diabetes easily, because of ageing, weakness of physical ability, or deficiency in immune system. However, although the common ageing factors in our study did not make much difference in changes during the follow-up period, they differed greatly in the risk of developing type 2 diabetes. In this regard, our findings can be emphasized by estimating the extent of type 2 diabetes risk affected by SHS exposure accumulated in adulthood, apart from the incidence of type 2 diabetes by general ageing.

A major strength of this research lies in that it is based on longitudinal data derived from a national representative sample of middle-aged adults over a 14-year period. This study is the first study that performed trajectory modeling about SHS exposure level using these data in Korea. Through the trajectory modeling of this study, we were able to explore qualitatively differentiated patterns of change during the follow-up period for SHS exposure levels. The trajectory modeling allowed a posteriori identification of qualitatively distinct trajectories, thus overcoming misclassification and loss of information. And also, we also expanded our study to include diabetes and impaired fasting glucose. The association of trajectories of SHS exposure with risk of type 2 diabetes or impaired fasting glucose was then examined using Cox proportional hazards models. Our findings provide important new insight regarding the trajectories of SHS exposure among middle-aged adults and their associations with the risk of diabetes. These results suggest that the most important thing to escape from exposure to SHS is not to be fully exposed to SHS from a young age. However, it might be important to investigate whether SHS trajectories in childhood also display consistent and similar heterogeneity and whether this heterogeneity has similar implications for diabetes risk.

This study has several limitations. First, our study has a lack of SHS exposure observations in early young adulthood. However, our results are reasonable in that we used the general population representing Korea to target specific generations vulnerable to chronic diseases from a life course perspective. Second, this study included the limited sample that can be used with a unique study design. Due to the nature of follow-up observational studies, many measurements that can be used in the study are often forced to give up due to the failure of the population’s follow-up observations. However, to correct the bias of loss to follow-up, we made the weighting by using the IPCW approach and applied the weighting to Cox proportional hazards models to estimate the effect of developing type 2 diabetes.^18 23^ The trajectory modeling also has the advantage of being able to function in missing data.^20^ Third, SHS exposure levels at work were not available due to a lack of available information on the base data. The number of respondents to the question of exposure to SHS at work was about 550 in baseline. In fact, since the most detailed data investigated on SHS exposure in Korea are the base data of this study, future research needs to further investigate and include SHS exposure at home, and patterns of individuals’ behavior, such as workplaces, and other places with high potential for SHS. Fourth, the limitation of generalization to other ethnic may exist, as a racially heterogeneous cohort of Asians. Finally, in the results of this study, causality is not guaranteed.

In conclusion, latent SHS exposure trajectories identified four distinct patterns during the follow-up period, which showed influence to development of a type 2 diabetes event. It provides new insights that the trajectory had a sharply decreasing pattern of SHS exposure up to a level of the lowest exposure pattern, but it had a higher risk of type 2 diabetes as a twofold compared with the lowest exposure pattern. Our study can suggest as an aspect of life course approach that even though resolving the external exposure of SHS in middle-aged adults, elevated SHS exposure level up to above 180 min/day in early adulthood may suffer glucose metabolic structure in a way that is not reversible. And also it can highlight the importance of SHS exposure prevention across life span, and prognostic assessments and targeted strategies for high-risk individuals by using trajectory modeling.
