# Supplementary material for: Changes in secondhand smoke exposure levels and risk of type 2 diabetes in middle age: the Korean Genome and Epidemiology Study (KoGES)
Source: BMJ Open Diabetes Res Care. 2019 Dec 8;7(1):e000859. doi: 10.1136/bmjdrc-2019-000859 (PMC6904168; doi:10.1136/bmjdrc-2019-000859)
Supplement: Supplementary data [file bmjdrc-2019-000859supp001.pdf]

Supplementary materials

" Changes of Second-hand smoke exposure levels and risk of type 2 diabetes in middle age: The Korean Genome and Epidemiology Study (KoGES)"

Jooeun Jeon[1, 2], Keum Ji Jung[2], **Heejin Kimm**[2‡], Sun Ha Jee[2]

[1]Department of Public Health, Graduate School, Yonsei University, Seoul, Korea

[2]Department of Epidemiology and Health Promotion, Institute for Health Promotion,

Graduate School of Public Health, Yonsei University

[‡] **Heejin Kimm, PhD, MD (Corresponding Author):**

Department of Epidemiology and Health Promotion, Graduate School of Public Health,

Yonsei University

50 Yonsei-ro, Seodaemun-gu, Seoul, 03722, Republic of Korea

Tel: 82-2-2228-1531; Fax: 82-2-365-5118; E-mail: heejink@yuhs.ac

Supplementary materials

Supplementary Table 1. Hazard ratios (95%CI) for risk of type 2 diabetes according to trajectories of Secondhand smoking exposure levels among women, N=1,798

	Unadjusted model*	Multivariable model[a]	Fully adjusted model[b]
	RR (95% CI)	RR (95% CI)	RR (95% CI)
Trajectories of Second-hand smoking exposure			
Low-stable	1.00	1.00	1.00
Moderate to low	0.75 (0.57-1.00)	0.65 (0.49-0.86)	0.91 (0.65-1.29)
Moderate	0.79 (0.49-1.26)	0.68 (0.42-1.10)	0.67 (0.38-1.18)
High to low	**1.63 (1.13-2.37)**	**1.69 (1.16-2.47)**	**1.78 (1.12-2.83)**
P for trend	**0.0104**	**0.0072**	**0.0642**

Abbreviation: RR, Risk ratio; CI, confidence interval

[a]Model Adjusted for age, family history of type 2 diabetes, alcohol drinking status, physical exercise, and body mass index, systolic blood pressure, total cholesterol level, educational levels, and household income at baseline

[b]Model Adjusted for Multivariable model + body mass index, systolic blood pressure, and total cholesterol level at endline

Jeon J, *et al. BMJ Open Diab Res Care* 2019; 7:e000859. doi: 10.1136/bmjdrc-2019-000859

Supplementary materials

Supplementary Table 2. Hazard ratios (95%CI) for risk of impaired fasting glucose according to trajectories of Secondhand smoking exposure levels among women, N=1,798

	Unadjusted model[a]	Multivariable model[a]	Fully adjusted model[b]
	RR (95% CI)	RR (95% CI)	RR (95% CI)
Trajectories of Second-hand smoking exposure			
Low-stable	1.00	1.00	1.00
Moderate to low	**1.30 (1.08-1.56)**	**1.24 (1.04-1.49)**	**1.35 (1.10-1.66)**
Moderate	**2.08 (1.63-2.66)**	**1.92 (1.50-2.46)**	**1.39 (1.06-1.82)**
High to low	**1.31 (1.00-1.72)**	**1.36 (1.03-1.78)**	**1.54 (1.14-2.07)**
P for trend	**0.0003**	**0.0003**	**0.0083**

Abbreviation: RR, Risk ratio; CI, confidence interval

[a]Model Adjusted for age, family history of type 2 diabetes, alcohol drinking status, physical exercise, and body mass index, systolic blood pressure, total cholesterol level, educational levels, and household income at baseline

[b]Model Adjusted for Multivariable model + body mass index, systolic blood pressure, and total cholesterol level at endline

Jeon J, *et al. BMJ Open Diab Res Care* 2019; 7:e000859. doi: 10.1136/bmjdrc-2019-000859

Supplementary materials

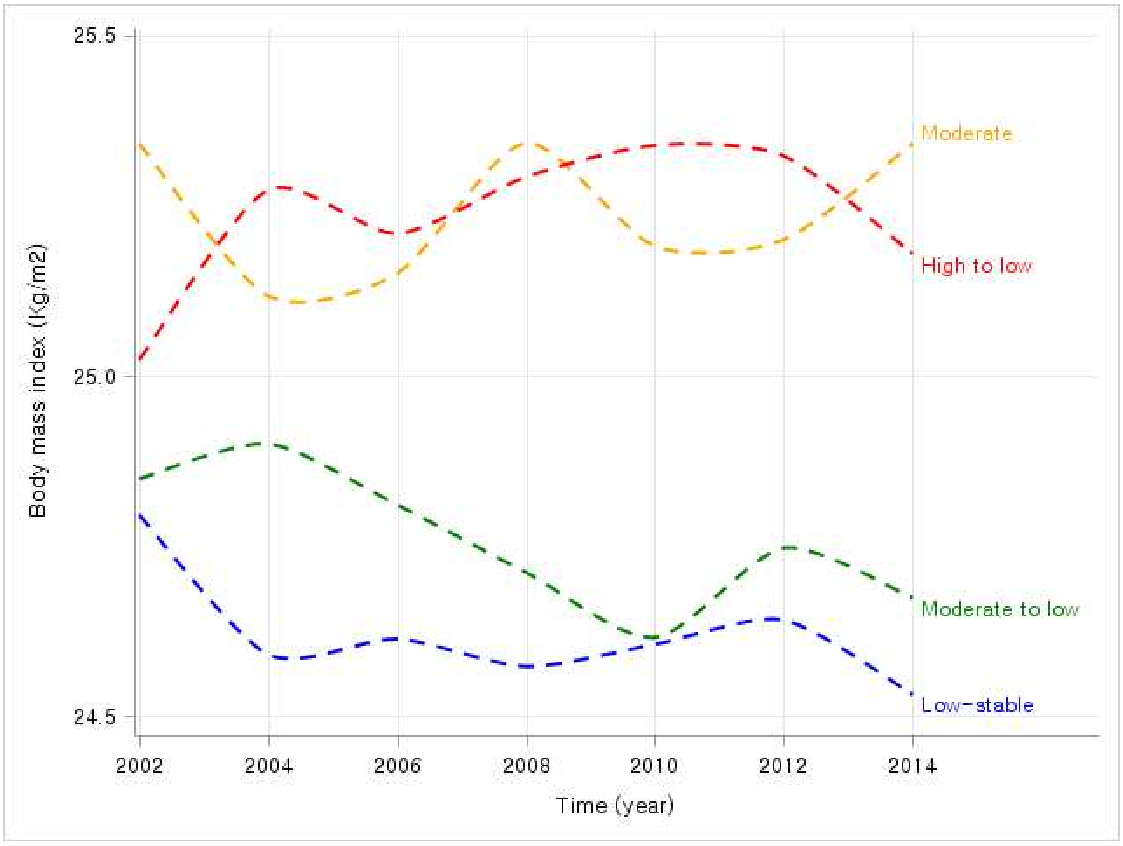

Supplementary Figure 1. Changes in body mass index during follow-up period by trajectory groups of secondhand smoking exposure levels

Jeon J, *et al. BMJ Open Diab Res Care* 2019; 7:e000859. doi: 10.1136/bmjdrc-2019-000859

Supplementary materials

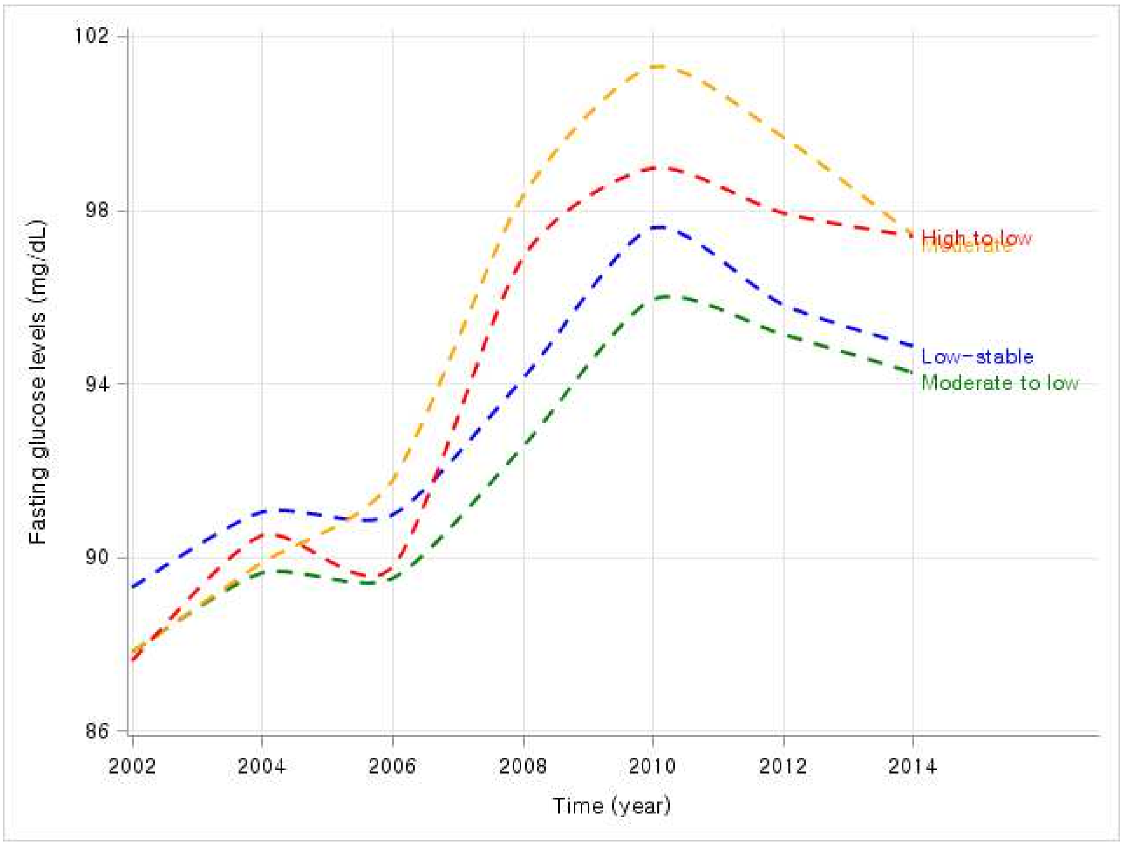

Supplementary Figure 2. Changes in fasting glucose level during follow-up period by trajectory groups of secondhand smoking exposure levels

Supplementary materials

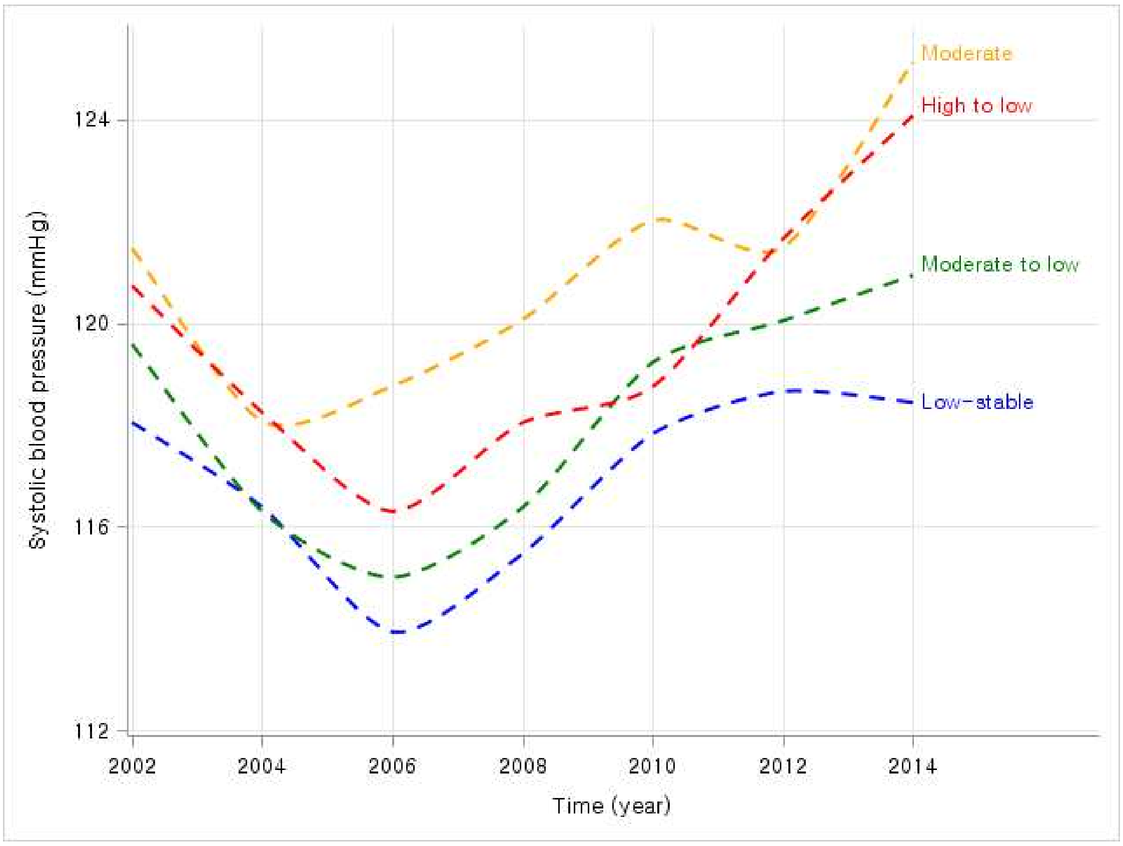

Supplementary Figure 3. Changes in systolic blood pressure during follow-up period by trajectory groups of secondhand smoking exposure levels
